# Evaluation of Printability of PVA-Based Tablets from Powder and Assessment of Critical Rheological Parameters

**DOI:** 10.3390/pharmaceutics16040553

**Published:** 2024-04-19

**Authors:** Jonas Lenhart, Florian Pöstges, Karl G. Wagner, Dominique J. Lunter

**Affiliations:** 1 Department of Pharmaceutical Technology, Eberhard Karls University, 72076 Tuebingen, Germany; jonas.lenhart@uni-tuebingen.de; 2 Department of Pharmaceutical Technology and Biopharmaceutics, University of Bonn, 53121 Bonn, Germany; florian.poestges@uni-bonn.de (F.P.); karl.wagner@uni-bonn.de (K.G.W.)

**Keywords:** 3D printing, hot-melt extrusion, personalized dosage, melt-rheology, dissolution, confocal Raman microspectroscopy, FabRX

## Abstract

Fused deposition modeling (FDM) is a rather new technology in the production of personalized dosage forms. The melting and printing of polymer–active pharmaceutical ingredient (API)—mixtures can be used to produce oral dosage forms with different dosage as well as release behavior. This process is utilized to increase the bioavailability of pharmaceutically relevant active ingredients that are poorly soluble in physiological medium by transforming them into solid amorphous dispersions (ASD). The release from such ASDs is expected to be faster and higher compared to the raw materials and thus enhance bioavailability. Printing directly from powder while forming ASDs from loperamide in Polyvinylalcohol was realized. Different techniques such as a change in infill and the incorporation of sorbitol as a plastisizer to change release patterns as well as a non-destructive way for the determination of API distribution were shown. By measuring the melt viscosities of the mixtures printed, a rheological model for the printer used is proposed.

## 1. Introduction

Three-dimensional printing as part of additive manufacturing has had huge impacts on different parts of technological advancements. With the beginning of research on the use of 3D printing in medical and pharmaceutical disciplines, the hopes for using the benefits of these techniques have risen. With Spritam^®^ and ZipDose^®^ technology (Aprecia Pharmaceuticals, LLC, Blue Ash, OH, USA), the first 3D printed tablet, using a combination of powder layering and inkjet technology, was approved by the FDA in 2015 [1]. Different extrusion-based technologies such as semi-solid extrusion (SSE) [2,3] and fused deposition modeling (FDM) were considered in the research of medical devices and pharmaceutical products, so much so that recently Triastek (Triastek, Inc., Nanjing, China) has received IND (Investigational New Drug) clearance for their second 3D printed product T20 using melt extrusion deposition (MED^TM^) technology [4]. With the rise of extrusion-ready pharmaceutical-grade excipients, tablets with different release patterns were produced using the FDM-based 3D printing technique. In terms of orally administered dosage forms, immediate release [5], modified release [6,7], bilayer [8], and even intra-gastric floating tablets [9,10] have been developed. The main advantage of 3D printed medicines is seen in the ability to produce customizable and patient-oriented geometries and strengths of tablets as well as in rapid prototyping [11]. While the general acceptability of 3D printing has been shown, concerns were found regarding different shapes. Already established shapes such as capsule or disc-shaped printlets are regarded as acceptable for swallowing, whereas more intricate shapes such as tilted diamonds were harder to “sell” to the testing group [7]. The first approaches of 3D printing used commercially available 3D printers and filaments. The filament was loaded with the API (active pharmaceutical ingredient) using different techniques such as loading by soaking [12]. The disadvantages found in the control of the loading and general concerns about solvent usage over time rendered this method obsolete. The next step was to extrude the filament needed directly from raw materials and to include the API in the desired concentration [13]. Filament production is challenging since an additional heating step is needed, which can negatively impact thermo-degradable drugs and polymers. Also, production must be strictly controlled to result in a sufficiently uniform filament. To avoid these problems, the extrusion and printing must take place in a single step. The company FabRx (FabRx Ltd., London, UK) developed a 3D printer especially for use in the development of pharmaceutical products. The filament printhead is exchanged for a printhead consisting of a small-scale single-screw extruder with a detachable nozzle which enables so-called direct powder extrusion (DPE) [14]. This opened the door to the printing of many of the excipients already developed for hot-melt extrusion (HME). While printing with filaments requires extensive knowledge of excipients and devices to produce filaments with relevant properties such as tensile strength, these parameters are not relevant when printing directly from the powder. Mechanical properties are key to printability for filament printers. A main prerequisite is longitudinal rigidity, which is shown by a high Young’s modulus while allowing no deformation or breakage during mechanical stress in printers feeding elements [15]. Thus, printing temperature can be reduced for the use of thermo-degradable drugs and polymers by incorporating plasticizers.

The aim of our research was to show that biopharmaceutics classification system (BCS) class II [16] drugs can be directly printed from powdered excipients into tablets with different strengths while forming amorphous solid dispersions (ASD). BCS class II compounds show low (water-) solubility and high permeability, so an improvement of the solubility is often searched for. Polyvinylalcohol (PVA) is a well-researched pharmaceutical excipient that shows appropriate thermal behavior (no thermal degradation up to at least 230 °C, appropriate melt viscosity) as well as water solubility [17]. As per the safety data sheet, the melting region is listed as 160–240 °C and the degradation temperature as above 200 °C. The synthetic opioid agonist loperamide (hydrochloride) [18] was used as a thermally stable BCS class II compound. One of the main challenges was to be able to form ASDs while performing the printing at temperatures well below the melting point of loperamide (220–228 °C [19]) since the thermal degradation of PVA starts below the melting temperature of loperamide. Information about the requirements of excipients for extrusion/3D printing using direct powder extrusion is scarce. Thus, a rheological model for PVA showing the minimum melt viscosity needed as well as the temperature and shear rate dependency was investigated.

Melt rheology and information about extrusion processes can be crucial for hot-melt extrusion as well as extrusion-based 3D printing. In previous studies, different approaches have been investigated to generate information regarding certain printers. Temperature as well as the closely related melt viscosity were parameters that were found to be of importance for further development [20,21]. Too low viscosity leads to oozing and dripping from the nozzle, reducing print quality, while too high viscosity leads to improper material flow up to a clogged nozzle or reduced layer adhesion.

Regarding the release behavior, the aim was set to be equivalent to typical formulations with immediate to sustained release.

## 2. Materials and Methods

### 2.1. Materials

PVA (Parteck^®^ MXP (Polyvinylalcohol), Merck KGaA, Darmstadt, Germany) was used as a water-soluble pharma-grade excipient available for (HME). Fumed silica (Aerosil^®^ R 972 Pharma, Evonik, Essen, Germany) was added to improve the flowability of the powder mixtures. As model API, loperamide hydrochloride (100% purity, Merck KGaA, Darmstadt, Germany) was selected. As a commonly used plasticizer for PVA, sorbitol (Parteck SI 400, Merck KGaA, Darmstadt, Germany) was used. All solvents used (Methanol, Acetonitrile, ammonium acetate) were HPLC-grade.

### 2.2. Methods

#### 2.2.1. Thermal Analysis

##### Differential Scanning Calorimetry (DSC)

Pure substances were analyzed via DSC (Mettler DSC 820, Mettler-Toledo GmbH, Gießen, Germany) regarding their melting point, glass transition temperature, and possible recrystallization. Printed tablets were measured after 2–4 weeks of storage. Approximately 10–15 mg samples were accurately weighed into sealed aluminum pans with punctured lids. The measurements used a heat-cool-heat cycle to determine the melting point in the first heating and glass transition temperature during the second heating. Information about the miscibility of loperamide (LOP) and Parteck MXP (PAR) was expected to be found during the DSC trials. One of the approaches for the estimation of glass transition temperature is described by the Gordon–Taylor equation, which can be applied to miscible blends.

(1) $$T_{g,\;mix}\approx \frac{\left[\omega_{1}*T_{g,\;1}+K*\omega_{2}*T_{g,\;2}\right]}{\omega_{1}+K*\omega_{2}}$$

with T_g,mix_ and T_g,i_ representing the glass transition temperature of the mixture and the components, ω_i_ is the mass fraction component I, and K is an adjustable fitting parameter.

It is expected for two miscible substances to show one glass transition temperature instead of two individual glass transition temperatures. The detected temperature should be partially composed of the individual glass transition temperatures [22].

##### Simultaneous Thermal Analyzer (STA)

Thermal degradation as well as the loss of water taken up from air humidity during printing and storage was tested using a Netzsch STA 409 PG/1/G Luxx (Erich NETZSCH GmbH & Co. Holding KG, Hanau am Main, Germany). All samples were treated the same way, measuring the mass of a sample using Al_2_O_3_ as a reference during the heating of the samples up to 250 °C. Pure substances and printed tablets were measured at a mass of approximately 22 mg.

#### 2.2.2. XRD-Measurements

Wide angle X-ray (powder) diffraction patterns were obtained in an angular range of 10–50° 2θ with a stepwise size of 0.02° on a Bruker D8 Advance diffractometer (Bruker Corp., Billerica, MA, USA) using monochromatic CuKα radiation (λ = 0.15406 nm). Pure substances were measured in powdered form, while printed tablets were measured intact.

#### 2.2.3. Melt Rheology

To measure the viscosity of the molten mixtures and thus link the rheological properties to the printing process, the shear rate occurring during printing was determined. Two different methods related to different extrusion processes were compared.

The first method is usually used in FDM 3D printers that run on filament. Here, only the shear rate in the nozzle is considered [23].

Volume flow (Q) was determined empirically by accurately weighing printed tablets with 100% infill and measuring their volume (V) using a gas displacement pycnometer (Accupyc 1330, Micromeritics Instrument Corporation, Norcross, GA, USA). The temperature was set to 25 °C while the remaining volume was flushed with Helium. During printing, the time (t) for each individual tablet was recorded. The following equation was employed afterwards:

(2) $$Q=\frac{V\;[mm^{3}]}{t\;[s]}$$

The apparent shear rate at the nozzle wall (${\dot{\gamma }}_{wa}$) can be calculated as:

(3) $${\dot{\gamma }}_{wa}=\frac{4Q}{\pi r_{n}^{3}}$$

with a radius (r) of the equipped nozzle of 0.04 cm.

The second method is applied to extrusion processes and used to calculate shear rates inside single-screw extruders. An approximation of the shear rate $\dot{\gamma }$ in the screw channel can be achieved from the Couette shear rate:

(4) $$\dot{\gamma }=\frac{v_{b}}{H}=\frac{\pi DN}{H}$$

with v (velocity), H (channel depth = 0.55 mm), D (diameter = 8.0 mm), and N (screw speed) [24].

Melt rheology was conducted using a compact rheometer (Anton Paar Physica MCR 501, Anton Paar GmbH, Ostfildern, Germany) equipped with single-use stainless steel plates with a radius of 10 mm in a plate–plate configuration. The sample specimens used for the melt rheology were produced using the MeltPrep device (MeltPrep GmbH, Graz, Austria) with a 20 mm disc geometry at temperatures of 10 °C above printing temperature, yielding ASDs with a consistent geometric shape [25]. Since the viscosity of non-Newtonian fluids is a product of the temperature and shear rate [26], the samples were measured at a range of temperatures after the determination of the linear viscoelastic region (LVR). The determination of the LVR was conducted for every measured temperature individually by performing amplitude sweeps from 0.01% to 100% deformation (0.0103 to 103 mrad) at 10 rad/s. Frequency sweeps from 100 Hz to 0.1 Hz were performed afterwards at 1% amplitude.

#### 2.2.4. HME of PVA/Sorbitol Mixtures

Apart from PAR/LOP mixtures, also plasticized mixtures were used. Sorbitol (SOR) was used as the plasticizer. The direct powder extrusion of PAR/SOR mixtures was not possible due to the different melting points. To be able to still print this mixture, a single screw extruder (Noztek Pro, Noztek, Shoreham-by-Sea, UK) was used to extrude these two excipients before adding the API and printing the mixture. Afterward, it was milled using an ethanol-cooled mill and sieved while the fraction < 0.400 mm was used in further printing steps.

#### 2.2.5. Preparation of Powder Mixtures

All powder mixtures for printing were produced by sieving (0.400 mm mesh size) and accurately weighing the compounds needed, adding Aerosil (AER) and subsequent mixing in a tumbler mixer (Turbula Type T2C, Willy A. Bachofen AG, Muttenz, Switzerland) for 20 min. Table 1 shows the composition of powder mixtures for printing.

#### 2.2.6. 3D Printing Using Direct Powder Extrusion Tool of M3dimaker

**Figure 1:**
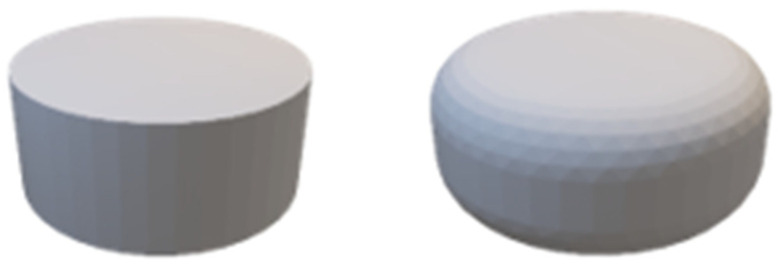
.stl file of tablet-shaped geometry (d = 5 mm; h = 5 mm) (**left**) and .stl file of improved geometry (d = 5 mm; h = 5 mm; edges rounded with r = 2 mm) (**right**).

A computer-aided design (CAD) tablet-shaped geometry was created using Autodesk^®^ Fusion 360 (Autodesk GmbH, München, Germany). The .stl file (seen in Figure 1) was sliced by Repetier-Host (V2.2.3) (Hot-World GmbH & Co. KG, Willich, Germany). Objects were printed with 2 outer perimeters and 3 bottom and top layers using varying infills to change the dosage of printed tablets on the M3dimaker 3D printer (FabRx Ltd., London, UK) equipped with the direct powder extrusion (DPE) tool. Print speed and temperature were adjusted according to the properties of the mixtures in a molten state.

The reason for choosing the rounded edges for further experiments was that the edges of the printed tablets were sharper than expected and needed to be rounded for more safety in handling.

#### 2.2.7. Confocal Raman Microspectroscopy

Witec’s alpha 300R system (WITec GmbH, Ulm, Germany) was employed for the measurements. Pure substances were measured with the 30 mW power of the laser (λ = 532 nm) and the evaluated spectra were used for the true component analysis to show the distribution of loperamide in the tablet.

Printed tablets were measured on the outer surface to find information on potential demixing processes, varying concentrations, and recrystallization on the outer surface.

One printed tablet of each mixture was measured using a 30 mW laser (λ = 532 nm). The surface of the measurement area was evaluated and corrected by the TrueSurface Mk II module. An area of 20 × 2000 µm with 4 × 400 pixels was measured using the autofocus function.

For the evaluation of drug content, the true component analysis integrated into the Witec Control 6 software was employed using the previously obtained spectra of individual substances. The software calculates based on individual spectra and the amount of drug found in each pixel measured and reports a colored picture of the distribution and intensity of the signal found.

#### 2.2.8. Drug Content Analysis by HPLC

An evaluation of the drug content was performed on the powdered mixtures as well as on the printed tablets. From the powdered samples, an equivalent of 5 mg of loperamide was accurately weighed and dissolved in 100 mL of the mobile phase used for the subsequent HPLC analysis (purified water/acetonitrile/0.5% ammonium acetate solution 29/36/35 (*v*/*v*)). Tablets were also dissolved in the mobile phase using stirring and an ultrasonic bath. The drug contents were determined by HPLC-UV/VIS (System 20A, Shimadzu Ltd., Kyoto, Japan) at a wavelength of 254 nm. The method showed a linearity of R^2^ = 0.99680 between 0.0023 mg/mL and 0.07 mg/mL with a tested LOQ of 0.00166 mg/mL and an estimated LOD of 0.0005488 mg/mL calculated according to ICH guidelines [27]. All experiments were performed in triplicate.

#### 2.2.9. In Vitro Dissolution

Dissolution testing was adapted from the standard procedures of the FDA [28] used for loperamide capsules using a Pharma Test PT-DT7 (Pharma Test Apparatebau AG, Hainburg, Germany) manual dissolution tester. First, 0.1 M of HCl was used as a dissolution medium (500 or 900 mL, depending on the tablet strength) at 37 °C with 100 RPM paddle speed. Sampling of 1 mL took place every 30 min for 5.5 h and a last sample was drawn after 24 h. The samples were measured using UV-HPLC as described in 2.2.8. For each formulation and infill setting printed, 4 tablets were measured and the mean value including the standard deviation is shown. For each formulation, a linear regression of the correlation between mass and infill is calculated and the best-fit values are given. Slopes are compared using two-tailed testing with the null hypothesis that the slopes are identical.

## 3. Results and Discussion

### 3.1. Results of Thermal Analysis

The thermal properties of the excipients, mainly the melting point and the glass transition temperature (DSC) and degradation (STA), were measured. From the powdered samples, information about the solubility was expected, while printed tablets were controlled for signs of crystallization of the API.

In the following diagrams, the thermograms of the measured samples (pure substances, powder mixtures, and printed tablets) are shown.

#### 3.1.1. Physical Characterization Using DSC

Figures showing the DSC data are contained in the supplementary part Figures S1 and S3, which show the curves of the first heating run where crystalline substances showed their melting point, while amorphous substances showed their glass transition temperature. The glass transition temperature of printed tablets was used as an indicator for the formation of ASDs. Similar Figures S2 and S4 contain the thermograms of the second heating run. The second heating run is used to determine the glass transition temperature of previously crystalline substances and information about the solubility of the API and polymer for powdered mixtures.

Table 2 gives an overview of the measured melting and glass transition temperatures.

During the first heating, the melting point of sorbitol and loperamide was detected as well as the semicrystalline nature of PVA indicated by a glass transition at 48–55 °C and a melting point of ∼180 °C. During the second heating run, the glass transition temperatures of sorbitol (−2.60–1.50 °C) and loperamide (51.04–63.16 °C) were found.

In the powdered samples, there were no signs of crystalline loperamide or sorbitol found even during the first heating where crystalline loperamide was contained. This indicates a good solubility of loperamide in PAR, at least at elevated temperatures. The missing individual glass transition of loperamide and sorbitol in the second heating indicates a miscible system formed from the API and the polymer and sorbitol, respectively. The change in Tg due to Loperamide may be only very slight, while it shows no second glass transition. It can still be seen that the measurement of powdered samples shows only the Tg of PVA in the first heating, while printed samples as well as the measurements of the second heating show a slightly lower Tg (than PVA in the second heating), which is more pronounced in the sample with a higher drug content. No clear glass transition temperature could be obtained from the Batch 3 printed samples over multiple runs; thus, no temperature was given.

The mixtures containing 15% sorbitol showed a decreased glass transition temperature (31.21–54.07 °C for the powder and 34.68–53.98 °C for the printlet), indicating that miscible systems were formed from PAR and SOR. In these thermograms, only one glass transition temperature was found. This temperature is composed of the two individual temperatures one would find for non-miscible systems.

Concluding the DSC experiments, sorbitol showed to be a good choice for use as a plasticizer and loperamide showed miscibility with the polymer in the measured concentrations. The results for sorbitol were expected, as its use as a plasticizer has been shown before [29,30].

#### 3.1.2. Results of Thermal Stability Using STA

**Figure 2:**
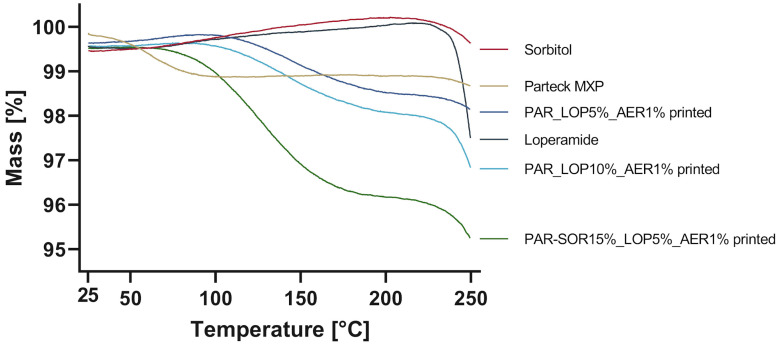
Change in mass during heating up to 250 °C.

The powdered samples were accurately weighed, while printed tablets were broken down and then weighed into the crucibles for analysis of thermal stability and potential water loss. The results are shown in Figure 2.

Significant mass loss was found for Parteck MXP (1%) as well as for the printed tablets. The mass loss in neat PAR could be attributed to solvent loss, as 1–3% methanol can be expected as per SDS. An increased mass loss was found for the sample containing 15% sorbitol, which might be a result of water uptake from humidity during printing or storage. An increase in sorbitol in PVA-based films has been shown to also increase its capacity to retain water, which is attributed to physically weak but chemically strong bonded water [29,30]. Loperamide and sorbitol were thermally stable up to at least 230 °C, as no degradation could be found.

### 3.2. Physical State Examination—XRD

The DSC measurements indicated the amorphous state of loperamide in PAR. To further harden this assumption, a second method to gain information about the physical state of the API at room temperature was implemented.

**Figure 3:**
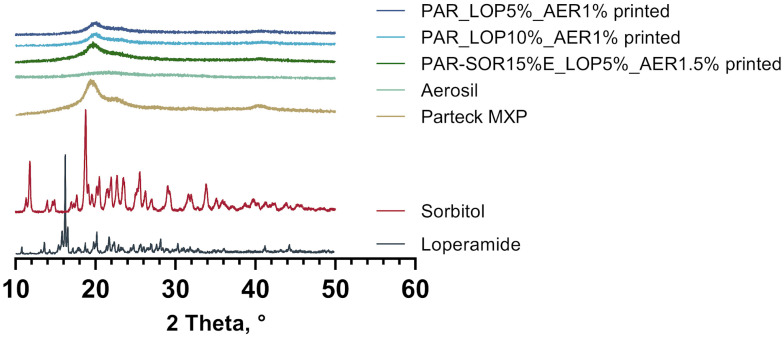
XRD spectra of pure substances and printed tablets.

Using XRD, whole printed tablets were measured, and the obtained spectra were compared to the spectra of pure substances. The results are given in Figure 3.

The spectra obtained from loperamide and sorbitol showed distinct peaks at a variety of angles, which was expected from crystalline substances. In the printed tablets, no signs of crystalline loperamide or sorbitol were found. The semicrystalline nature of PAR was found in the pure PAR as well as the printed tablets. This complies with the results from thermal analysis, underpinning the amorphous nature of loperamide in the printed tablets. Although the amorphous halo is widely regarded as a sign of absent crystalline structures, it can be found in other instances that include disordered nanocrystalline phases as well as glassy or amorphous ones [31].

### 3.3. 3D Printing

#### 3.3.1. PVA without Plasticizer

Apart from the suitable temperature regarding melt viscosity, there are further parameters of interest for extrusion using a small-scale single-screw extruder. Powder flowability is a key characteristic since the powder must flow into the barrel before it can be melted and pushed through. As the mixtures showed insufficient flowability, adjustments were made by adding 1% of AER to ensure sufficient flow behavior.

**Figure 4:**
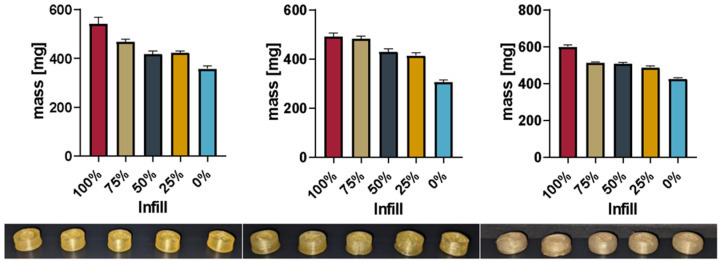
Masses of printed tablets Batch 1–3 (fltr) and printed tablets from corresponding batch (100–0% Infill fltr) in the individual pictures.

The direct printing of Batch 1 (Table 1) was successful using a 200 °C nozzle temperature and 40 °C print bed temperature. Tablets of different strengths were produced by varying the infill in 25% increments (100%, 75%, 50%, 25%, 0%). In the next iteration, the drug load was increased by doubling the API concentration in the powder mixture (Batch 2, Table 1). Printing was successful at 205 °C/40 °C, with tablets printed from 100–0% infill in 25% increments. Figure 4 shows the average mass including the standard deviation of the printed tablets as well as pictures of one tablet from each infill (FLTR: 100–0% Infill). To enhance understanding of the infill procedure, sliced pictures of the tablets can be found in Figure S5.

#### 3.3.2. PVA with 15% Sorbitol

To decrease the dissolution times and reduce the printing temperature, 15% sorbitol was added to the PAR. Batch 3 (Table 1) was successfully printed using 185 °C/40 °C to print tablets varying in infill, as seen inthe pictures in Figure 4. Figure 4 shows the mass and standard deviation of the printed tablets and exemplary pictures of the tablets from each infill percentage using the updated shape.

**Figure 5:**
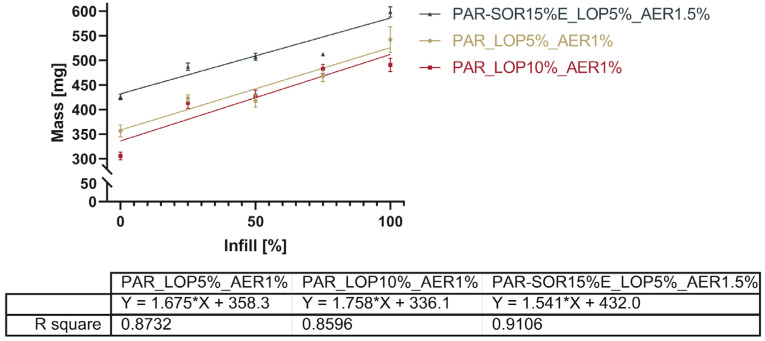
Linearity of the relation between set infill and measured mass.

Figure 5 gives a comprehensive overview of the mass of all printed tablets.

The total mass of the printed tablets showed a reduction with the reduced infill, as expected. Figure 5 shows the relation between the infill set in the slicing software and the actual mass of the printed tablets.

The linearity of the correlation between the infill and total mass (as shown in Figure 5) is found to be between 0.8596 and 0.9106. A comparison of the slopes by analysis of the covariances showed no significant difference between the slopes (*p* = 0.5053). This indicates that it is possible to dictate the drug load from the infill independent of the powder mixture used. The slicing software determined based on the shape of the object and nozzle size at which position additional material would be deposited. Differences between 25%, 50%, and 75% were not as predictable as the software suggested, e.g., the difference in the mass between the tablets with 25% and 50% infill was way smaller than the correlation seemed to predict. Stepping forward to 3D printing from material evaluation such as melt rheology, one has to keep in mind that being able to extrude material does not necessarily mean that it is the right setup for printing as well. Successful printing, unlike extrusion, does not only depend on material being able to push from the nozzle; sometimes, the adhesion of the print bed as well as the adhesion of different layers need an increase or decrease in temperature for the successful bonding of the layers. Therefore, subtle differences in printing temperatures were found to be necessary.

### 3.4. Measurement of Rheological Properties of the Mixtures in Molten State

The determination of the shear rate was conducted using tablets consisting of PAR-SOR15%E/LOP5%/AER1% printed at 185 °C and 6 mm/s print speed. The print speed represents a consideration between decreasing the time needed to print a tablet and the torque capacity of the motor as well as finding a temperature where material could be printed without oozing from the nozzle.

Individual printing times of 558 ± 2.45 s were measured for a batch of five consecutive printed tablets. During this time, 15.8 screw turns were recorded and an actual volume of the printlet of 0.461 ± 0.005 cm^3^ was measured, compared to the theoretical volume of 0.4936 cm^3^.

This results in a volume flow Q of 8.26 × 10 − 4 ± 7.80 × 10^−6^ cm^3^/s, which gives a shear rate in the nozzle of 16.43 ± 0.155 1/s.

For the same tablets, the couette shear rate was calculated using the aforementioned dimensions of the extruder screw used. With an N (screw rotational speed) of 2.83 × 10^−2^ ± 1.24 × 10^−4^ 1/s, a Couette shear rate of 1.294 ± 5.68 × 10^−3^ 1/s was calculated. The calculated shear rates were intended to be used in combination with the rheological data to make a claim about the viscosity of a polymer melt suitable for printing.

**Figure 6:**
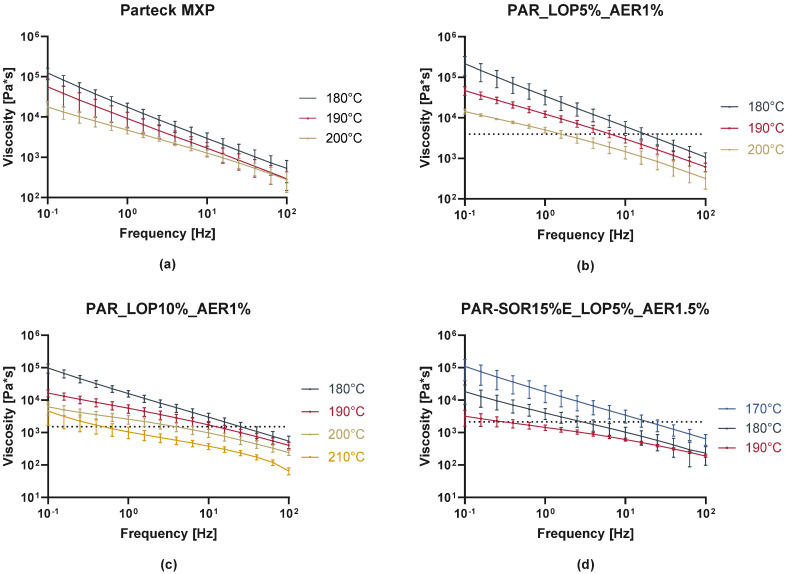
Melt viscosity of neat polymer and printed formulations. (**a**) shows the curves obtained from neat PAR, while (**b**–**d**) show the curves obtained from the three printed batches, respectively.

In rheologic measurements, the linear viscoelastic range (LVR) was determined via amplitude sweeps. A deformation of 1% was found to be in the LVR for all the samples at the measured temperatures. Afterwards, frequency sweeps from 100 to 0.1 Hz were performed at a temperature around the printing temperature (Figure 6). As expected, the viscosity decreased with the increasing temperature, increasing frequency, and with the addition of the plasticizer sorbitol. Prior research indicates that usually viscosities from 100,000 to 10,000 Pa·s are found during HME processes [32]. Maximum viscosities as seen in Figure 6 needed at the lower calculated shear rate (Couette: 1.294 1/s) were found at ranges from 1500 to 4000 Pa·s, which indicates that a rather low melt viscosity is needed to print successfully with the M3dimaker. The dashed lines represent the viscosity of the mixture at the calculated shear rate and temperature used to print.

This correlates well with the fact that compared to lab-scale extruders, this printer comprises a very small screw and a motor of lower torque to keep the weight down and increase the precision of the printing. We propose that the selection of polymer and its expected printing temperature should include frequency sweeps with increasing temperature until a viscosity of a maximum of 5000 Pa·s is measured. Similar experiments to find information about melt viscosity and temperature dependence have been conducted for other APIs and different use cases. They are often found under the name of “small amplitude oscillatory shear” (SAOS) experiments. It was shown that vacuum compression modeling (VCM) is a useful tool for the simulation of extruded samples in order to correctly predict extrusion parameters. Also, temperature as a crucial parameter for printability was predicted by melt rheology experiments [20,33].

### 3.5. Confocal Raman Microspectroscopy

**Figure 7:**
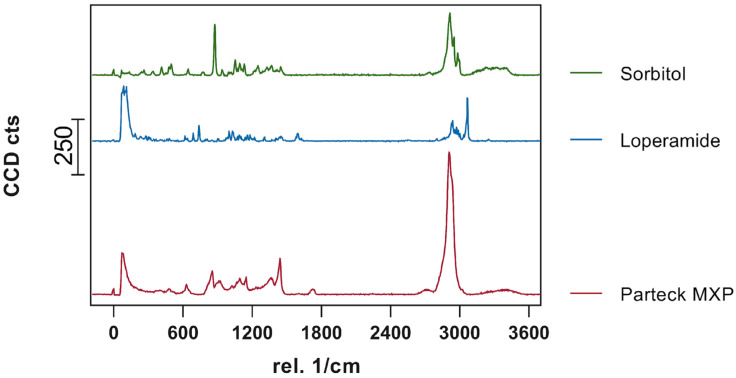
Obtained single spectra (0.5 s; 10 Acc.) red = Parteck MXP, blue = loperamide, green = sorbitol.

The visualization of API content, distribution, and potential recrystallization can be achieved via confocal Raman microspectroscopy (CRM). The overlay of the single spectra of LOP, SOR, and PAR (Figure 7) showed peaks that can be used for the evaluation of contained API in the printed tablets. The peak in the high wavenumber range (3040–3090 1/cm) as well as the peak in the fingerprint region (1575–1615 1/cm) have no significant overlapping with the peaks from other substances and can be used to identify loperamide. The low drug load of 5 and 10%, respectively, lead to a decreased signal intensity of these peaks in the resulting spectra of printed tablets relative to the single spectra obtained from pure substances.

**Figure 8:**
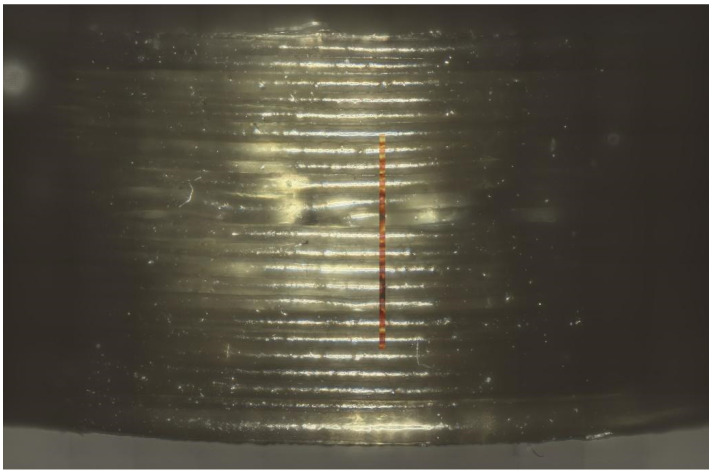
Picture of tablet printed from PAR_LOP5%_AER1% with an overlay of the measured area by true surface model.

Figure 8 shows an example of the measured area on the side of the printed tablet. The true surface module was able to cover most of the differences in the height of the surface area. Fine-tuning to achieve good spectra was completed via auto-focus.

**Figure 9:**
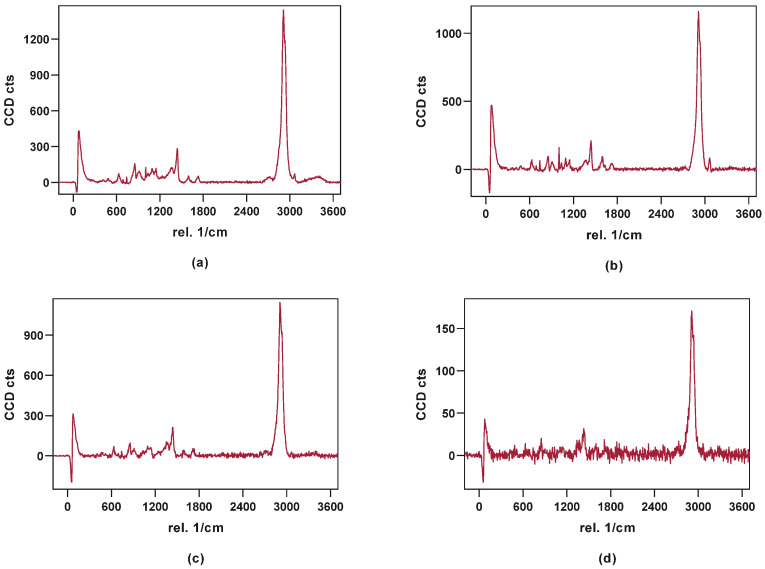
Exemplary spectra obtained from the measured area: (**a**) PAR_LOP5%_AER1%, (**b**) PAR_LOP10%_AER1%, (**c**) PAR-SOR15%E_LOP5%_AER1%, (**d**) failed focus or bad spectrum found in the light and blue areas as seen in Figure 10.

**Figure 10:**
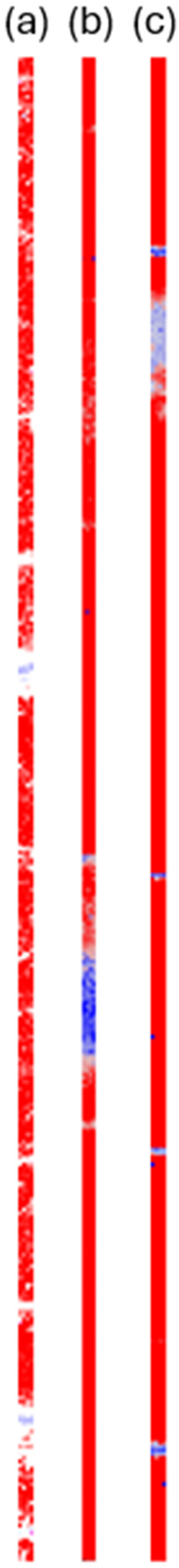
Distribution of loperamide based on the true component analysis is shown for all three printed tablets. (**a**–**c**) showing the distribution of tablets made from Batch 1, 2, and 3 respectively.

Exemplary spectra (Figure 9) were given for the individual colors used in the color coded pictures (Figure 10). Red shows the signal where loperamide was found and spectrum intensity was sufficient. With the loss in signal intensity (as seen in the lighter and blue-colored regions), the signal-to-noise ratio of the loperamide peaks get worse, although loperamide concentration remains constant.

Raman imaging was able to show consistent spectra containing polymer and API over a range of points distributed over roughly 40% of the printing time. No “hotspots” or crystalline API were found during these measurements, which supports findings from the XRPD measurements of a uniform ASD.

### 3.6. Drug Content

Since the powder mixtures contained 5% (m/m) and 10% of API, respectively, the expected drug content was calculated as 5% and 10%, respectively, of the total mass of the printed tablets. The actual drug content was assessed to make sure the correct amount was referred to as 100% release during in vitro measurements. By dissolving the whole tablet in a suitable medium, the actual drug content was determined. The results of this experiment are given in Table 3.

The drug content shown in Table 3 deviates from the expected amount. The deviations in content from the expected amount in the powdered samples can be attributed to adhesion to the mixing vessels and spatulas used. The reduction in the measured content of the printed tablets can partially be explained by the water uptake, which increases the determined total mass of the tablet without adding further API. Also, adhesion to the screw in the extruder is a possible explanation. Other reasons could be found in the demixing processes during printing, imperfect powder homogeneity, and adhesion to the glass and plastics of tubular glasses during mixing. Small batch sizes as well as small sampling sizes increase the errors found. Deviation regarding the drug content has been shown in previous studies as well [20,34,35,36], which might be reduced by in-line quality control mechanisms.

### 3.7. In Vitro Dissolution Testing of Printed Tablets

**Figure 11:**
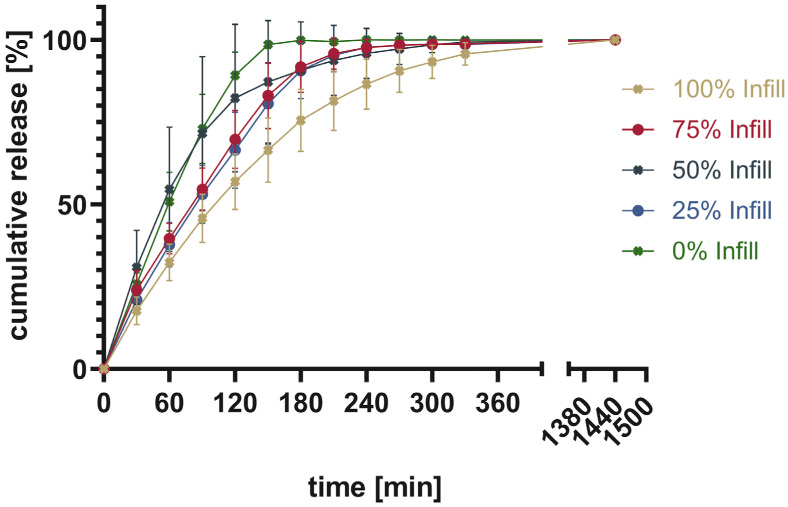
Cumulative release given in percentage of calculated drug load for PAR_LOP5%_AER1%.

**Figure 12:**
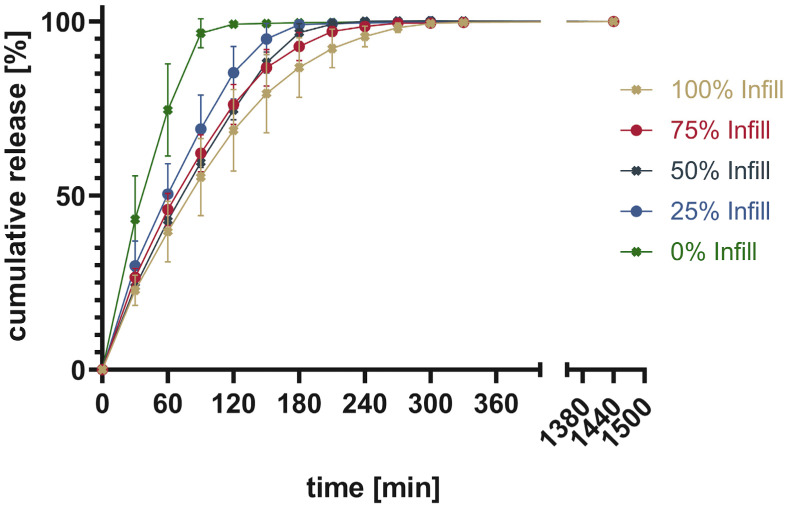
Cumulative release given in percentage of calculated drug load for PAR_LOP10%_AER1%.

**Figure 13:**
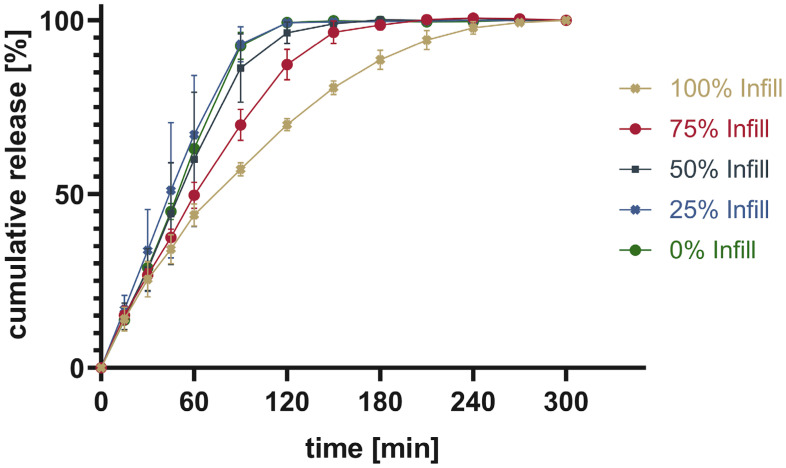
Cumulative release given in percentage of calculated drug load for PAR-SOR15%E_LOP5%_AER1%.

Four tablets were tested at once to show the average ± SD. The total drug content was calculated from the mass of the printed tablet as well as the measured content shown in Table 3. The cumulative released drug was calculated and put into relation to the measured content to give the percentage released. Figure 11 shows the percentage of cumulative drug release from PAR_LOP5%_AER1%, while Figure 12 and Figure 13 show the release of PAR_LOP10%_AER1% and PAR-SOR15%E_LOP5%_AER1%, respectively.

The release patterns of loperamide from printed PAR or PAR/SOR mixtures show that the higher the percentage infill of the tablets, the slower the release. This matches the expectations based on existing research [37]. Unexpectedly, overall, a very slow release was found for PAR without a plasticizer. This indicates that the incorporation of a poorly water-soluble drug into PAR resulted in a slower release. The addition of 15% sorbitol improved the dissolution time of the printed tablets independent of the infill. While for 100% infill, there was no difference between Batch 2 and 3, as both dissolved faster than Batch 1. For 75% and 50%, both Batch 1 and 2 showed no real difference in the dissolution time, while Batch 3 showed the fastest dissolution. For 25%, it can be observed that the dissolution time decreases from Batch 1 to Batch 3 with Batch 2 in between. Finally, for 0% infill, both Batch 2 and 3 showed an improved dissolution rate over Batch 1. Increasing the drug load which leads to a reduction in polymer already improved the dissolution rate slightly. Still, the amount of infill showed a larger impact on the dissolution time than the addition of the highly water-soluble plasticizer. This can be explained by the different infill resulting in changes in the area-to-volume ratio. As soon as the outer layers of the tablet are penetrated by media, tablets with lower infill offer a larger area, and thus, the dissolution rate increases. Additionally, there were large standard deviations observed in the earlier sampling points. They can be attributed to the deviation of the dissolution behavior of individual tablets. These results are not surprising as tablets generally and 3D printed tablets especially do not always show very narrow dissolution profiles [38,39,40]. Thus, usually, only three sampling points are used to assess if the dissolution behavior is sufficient. One is used to secure against dose dumping, one intermediate to show control over the dissolution profile, and a final one to show full release [41]. Individual dissolution curves can be found in the supplementary, divided into each infill percentage (Figure S6).

## 4. Conclusions

Direct powder extrusion and the 3D printing of tablets was achieved with formulations containing PAR, sorbitol, and loperamide using the M3dimaker 3D printer. In printed tablets, loperamide appeared to form amorphous solid dispersions as characterized by DSC and XRPD. Confocal Raman microspectroscopy showed a homogenous distribution of the API during the print, underlining this hypothesis. Nevertheless, one has to keep in mind the limitations of the methods used. While CRS has a large spatial resolution of 5 μm, DSC is unable to detect phase-separated domains smaller than 30 nm. This makes a differentiation between amorphous solid dispersion and nanodispersion very tough [42,43]. The use of the two methods (DSC and XRD) is widely acknowledged for being able to resolve mistakes when looking for a solid-state characterization and confirmation of an amorphous state. Such includes increased solubility at elevated temperatures, as mentioned in the DSC results part [44,45,46]. The resolution of XRD is expected to be found at approx. 1% [47,48]. By employing three complimentary state-of-the-art methods, we can conclude that to the best of our knowledge and to the best of the current methods’ capabilities, the drug is present as ASD in the printed tablets.

The printing temperature was sufficiently low to encounter no thermal degradation of API, polymer, or other excipients. By adding the API after extruding the other excipients, one heating step and therefore the potential thermal degradation for the API could be eliminated. Control over the drug content was realized by changing the amount of infill. While this is a proven way to change drug content, one must keep in mind that changes in the area/volume ratio also can impact the release behavior. If the acceleration of the dissolution time is aimed for, the addition of (super)disintegrants could be a promising approach.

From the characterization of the melt properties of the mixtures, a system for the selection of polymers is proposed. Extrudability as well as printability seem to work sufficiently with a melt viscosity below 4000 Pa·s. This value could be used in future trials to reduce material use during preliminary tests. The range of usable polymers and even working temperatures for these polymers could be evaluated by conducting rheological tests.

## Figures and Tables

**Table 1:** Physical mixtures for 3D printing.

**Batch**		Parteck MXP	Parteck MXP/Sorbitol 15% Extrudate	Loperamide	Aerosil
1	PAR-LOP5%-AER1%	94.0%		5.0%	1.0%
2	PAR_LOP10%_AER1%	89.0%		10.0%	1.0%
3	PAR-SOR15%E_LOP5%_AER1%		93.5%	5.0%	1.5%

**Table 2:** Comprehensive results of glass transition temperatures found during DSC experiments.

Glass Transition First Heating	Onset [°C]	Endset [°C]	Glass Transition Second Heating	Onset [°C]	Endset [°C]
PAR	49.64	52.99	PAR	61.11	72.76
SOR	crystalline, Mp: 89.75 °C		SOR	−2.60	1.50
AER	N/A		AER	N/A	
LOP	crystalline, Mp: 227.47 °C		LOP	51.04	63.16
Batch 1 powder	47.11	52.35	Batch 1 powder	62.09	73.44
Batch 1 printed	56.44	60.79	Batch 1 printed	58.67	74.56
Batch 2 powder	44.33	48.40	Batch 2 powder	58.43	75.10
Batch 2 printed	56.07	61.85	Batch 2 printed	54.53	71.96
Batch 3 powder	N/A		Batch 3 powder	31.21	54.07
Batch 3 printed	N/A		Batch 3 printed	34.68	53.98

**Table 3:** Loperamide content in powdered and printed samples.

	PAR_LOP5%_AER1%		PAR_LOP10%_AER1%		PAR-SOR15%E_LOP5%_AER1%	
	Powdered	Printed	Powdered	Printed	Powdered	Printed
Mean API content	92.63%	95.13%	88.49%	82.99%	95.85%	85.40%
Standard deviation	2.74%	12.88%	1.50%	1.30%	5.65%	0.92%

## Data Availability

Data is contained within the article or Supplementary Materials.

## Supplementary Materials

The following supporting information can be downloaded at: https://www.mdpi.com/article/10.3390/pharmaceutics16040553/s1, Figure S1: First heating neat substances; Figure S2: Second heating: neat substances; Figure S3: First heating: powdered and printed samples; Figure S4: Second heating: powdered and printed samples; Figure S5: Sliced tablets for visualization of infill; Figure S6: Dissolution date of individual tablets (100%: (a), 75%: (b), 50%: (c), 25%: (d), 0%: (e)).
