# Supplementary material for: Evaluation of Printability of PVA-Based Tablets from Powder and Assessment of Critical Rheological Parameters
Source: Pharmaceutics. 2024 Apr 19;16(4):553. doi: 10.3390/pharmaceutics16040553 (PMC11054527; doi:10.3390/pharmaceutics16040553)
Supplement: Supplementary file 1 [file pharmaceutics-16-00553-s001.zip › pharmaceutics-2936397-supplementary.pdf]

pharmaceutics

MDPI

Supplementary

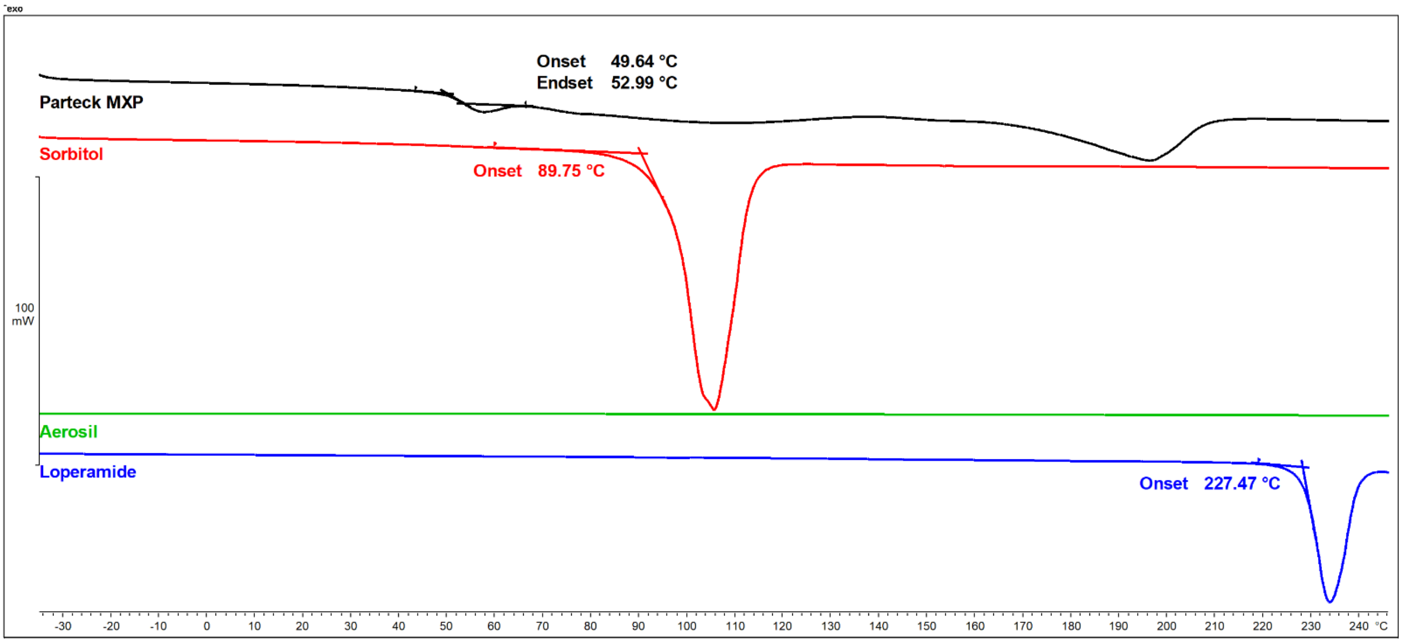

Figure S1. First heating: neat substances

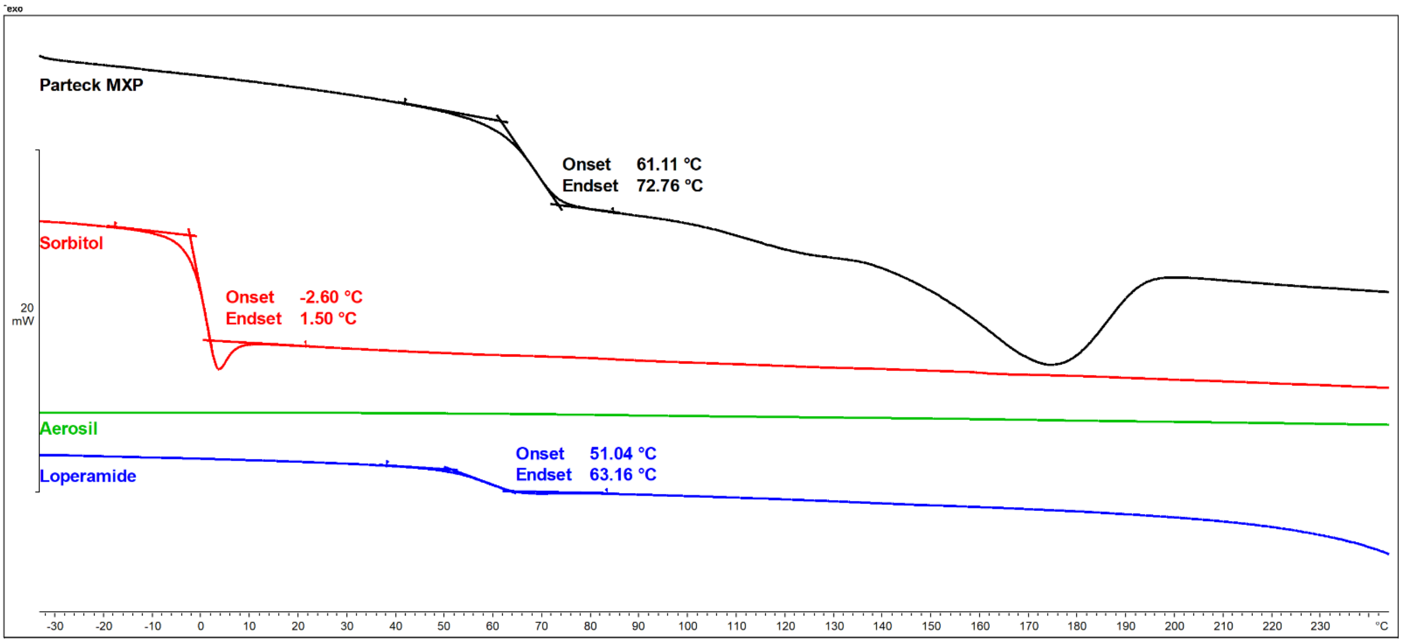

Figure S2. Second heating: neat substances

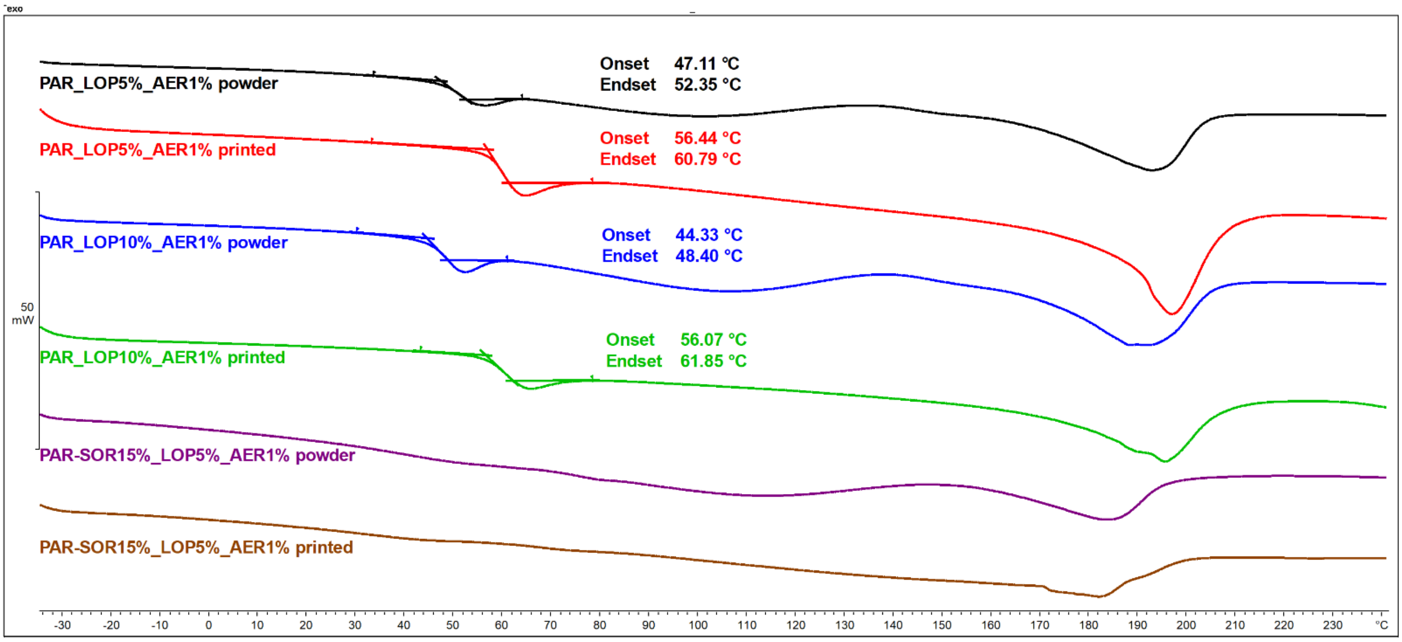

Figure S3. First heating: powdered and printed samples

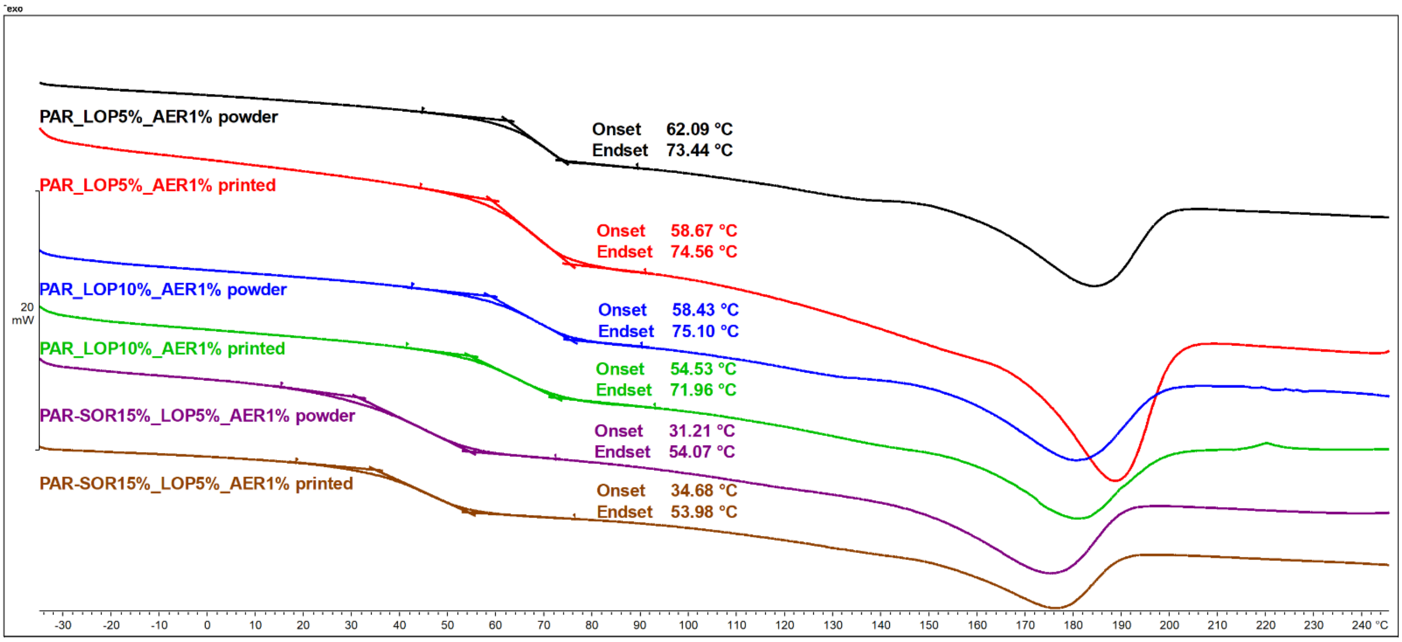

Figure S4. Second heating: powdered and printed samples

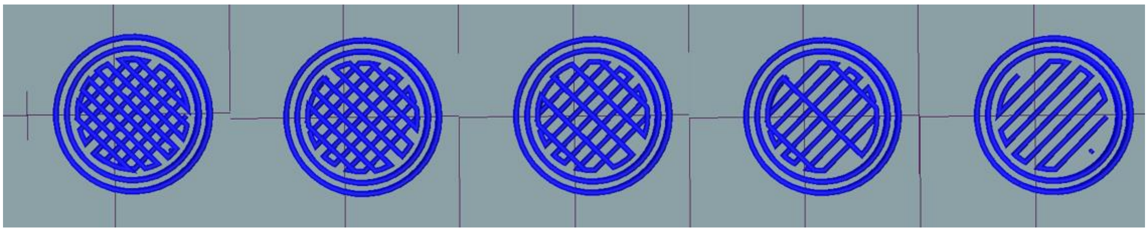

Figure S5. Sliced tablets for visualization of infill

The picture of the sliced tablets shows the layers 2-4 for each individual infill (100% - 0% fltr.) As shown above, the solid layer is constant for each infill and no additional infill is placed for 0% tablets. The addition of 3 top solid layers closes the hollow tablet sufficiently.

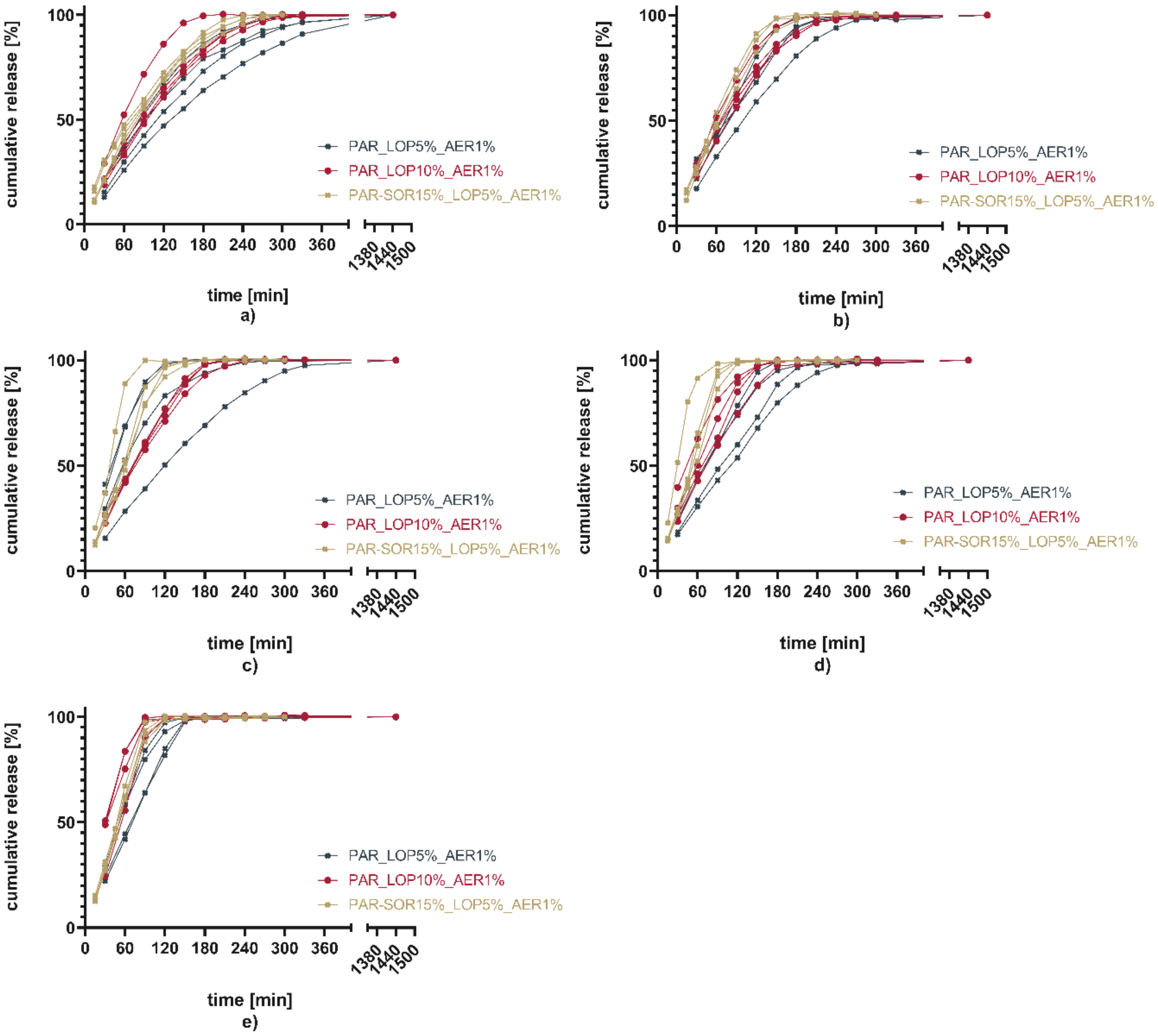

Figure S6. Dissolution data of individual tablets (100%: a), 75%: b), 50%: c), 25%: d), 0%: e))
